# Symbiotic System Establishment between *Piriformospora indica* and *Glycine max* and Its Effects on the Antioxidant Activity and Ion-Transporter-Related Gene Expression in Soybean under Salt Stress

**DOI:** 10.3390/ijms232314961

**Published:** 2022-11-29

**Authors:** Depeng Zhang, Xinsheng Wang, Zhenyue Zhang, Chunxin Li, Yimei Xing, Yaqin Luo, Donghuan Li, Zhiyun Ma, Hua Cai

**Affiliations:** College of Life Science, Northeast Agricultural University, Harbin 150030, China

**Keywords:** *Glycine max*, *Piriformospora indica*, antioxidants, salt tolerance, ion transporter relative genes

## Abstract

The utilization of symbiosis with beneficial microorganisms has considerable potential for increasing growth and resistance under abiotic stress. The endophytic root fungus *Piriformospora indica* has been shown to improve plant growth under salt and drought stress in diverse plant species, while there have been few reports of the interaction of *P. indica* with soybean under salt stress. In this study, the symbiotic system of *P. indica* and soybean (*Glycine max* L.) was established, and the effect of *P. indica* on soybean growth and salt tolerance was investigated. The colonized and non-colonized soybeans were subjected to salt stress (200 mmol/L NaCl), and the impairments in chlorophyll and increasing relative conductivity that can be caused by salt stress were alleviated in the *P. indica*-colonized plants. The accumulation of malondialdehyde (MDA), hydrogen peroxide (H_2_O_2_), and superoxide anion (O_2_^−^) were lower than that in non-colonized plants under salt treatment, whereas the activities of antioxidant enzymes were significantly increased by *P. indica* colonization, including superoxide dismutase (SOD), peroxidase (POD), catalase (CAT), and glutathione reductase (GR). Importantly, without salt treatment, the Na^+^ concentration was lower, and the K^+^ concentration was higher in the roots compared with non-colonized plants. Differential expressions of ion transporter genes were found in soybean roots after *P. indica* colonization. The *P. indica* colonization positively regulated the transcription level of *PM H^+^-ATPase*, *SOS1*, and *SOS2*. The study shows that *P. indica* enhances the growth and salt tolerance of soybean, providing a strategy for the agricultural production of soybean plants in saline-alkali soils.

## 1. Introduction

More than 800 million hectares of arid and semi-arid areas on Earth are affected by soil salinization and soil degradation, which seriously affects agricultural production and the ecological environment [1]. Excessive salinity in the soil can cause enormous damage to plant growth and reproduction and can also lead to reduced quality and yield, especially for crops. Furthermore, most crops and forage used in modern agriculture are salt-sensitive plants. Therefore, revegetating salinized land and enhancing the salt tolerance of plants are urgent problems that need to be solved.

Increasing numbers of reports suggest that root symbiotic microorganisms can enhance nutrient uptake, plant biomass, and yield [2], and more importantly, symbiotic interactions between host plants and some of their endophytic microorganisms can improve host plant tolerance to various environmental stresses [3,4,5]. Among these beneficial microorganisms, *Piriformospora indica* (also known as *Serendipita indica*) is one of the most talked about members. *P. indica* is a root endophyte that can aseptically cultivate and colonize plant roots. *P. indica* has almost all the beneficial properties of arbuscular mycorrhizal fungi (AMF) and has a wider plant host range, including model plants, such as Arabidopsis [6,7], tobacco [8], rice [9], and barley [10]. Furthermore, *P. indica* promotes growth-stimulating functions and resistance to various abiotic stresses, including drought, heavy metals, cold, and salt [11].

Many studies have shown that root symbiosis in *P. indica* mainly triggers the activation of antioxidant enzymes to decrease the accumulation of ROS in response to abiotic stress [12,13,14,15]. For instance, Dan Li et al. thought that photosystem efficiency, antioxidant enzymes, osmoprotectants, and cold-responsive genes played an essential beneficial role in *P. indica*-induced cold resistance in bananas. In other research [16], it was found that CAT and GR are two major targets of the fungus in rice seedlings under water stress. Under high-salinity stress, Khalid found *P. indica* co-culture significantly increased antioxidant enzymes such as SOD, POD, and CAT, which played a positive role in plants' response to salt stress [17].

In response to sodium toxicity, maintenance of Na^+^ and K^+^ ion homeostasis is an important defense mechanism developed by plants, including upregulating the expression of membrane ion transporter genes responsible for sodium transport and compartmentalization in plant cells [18]. It has been reported that *P. indica* can alleviate salt stress damage by modulating the Na^+^/K^+^ ratio of colonized plants in addition to altering antioxidant enzyme levels and inducing ROS scavenging systems [10]. In *Gerbera jamesonii*, *P. indica* colonization positively regulated the *NHX2* and *SOS1* transcription levels of genes involved in ionic homeostasis after salt treatment. Similarly, under salt stress, the Na^+^/K^+^ ratio of *P. indica*-colonized tomatoes was lower than that in corresponding non-colonized plants, and this change in ion homeostasis was accompanied by an increase in *LeNHX1* transcripts in the leaves of colonized tomatoes [19]. Khalid et al. quantified the salt tolerance mechanism of *P. indica*-inoculated pak choi by measuring the expression level of *SOS1*, *SOS2*, and NHX1 genes in the salt oversensitivity (SOS) signaling pathway. The gene was expressed at higher levels in the inoculated plants under salt stress [17].

Soybean (*Glycine max* L.) is an important oil crop and is widely cultivated. Soybeans, however, are moderately sensitive to salt, depending on variety and environmental characteristics [20]. Research on the salt tolerance of soybean has been gradually deepened in the past ten years; there are in-depth studies at the molecular, physiological, and biochemical levels [21] and on the interaction between plants and microorganisms [22]. Furthermore, it had been suggested that *P. indica* can colonize soybean roots [23], but the salt and alkali tolerance of *P. indica*-colonized soybean plants had not been reported. So, it is necessary to study the mechanism of salt tolerance in soybeans inoculated with *P. indica.* The aims of this research were (1) to establish the symbiotic system between *P. indica* and *Glycine max* to obtain sustainable and vigorous *P. indica* species resources, (2) to elucidate whether *P. indica* symbiosis affects the activation of antioxidant enzymes and the accumulation of ROS, Na^+^, and K^+^ uptake under salt stress, and (3) to highlight the mechanisms involved in the enhanced salt tolerance in *P. indica* co-cultivated soybean as an economically important plant. Through this study, we tried to promote the salt tolerance of soybeans through the interaction between microorganisms and plants and to provide a more economical method for improving soybean yield in saline-alkali land.

## 2. Results

### 2.1. Establishment of the Symbiotic System between Piriformis indica and Soybean

Different medium components have different effects on the growth of fungi and their colonization in plant roots [24]. In order to obtain efficient colonization in soybean roots, three media—Hoagland, PNM, and 1/2 MS—were investigated to establish a symbiotic system. After seven days of culture in the three media, *P. indica* showed some differences in growth (Supplementary Figure S1A). On PNM and Hoagland medium, *P. indica* covered almost the entire dish (growth radius of about 3.5 cm), while on 1/2 MS medium, the growth radius was only about 0.7 cm. It could be seen that Hoagland and PNM media were more suitable for the in vitro culture of *P. indica*. Some studies have shown that colonization efficiency decreases significantly with the increase of the algebraic expansion in culture. Rejuvenation of *P. indica* is an effective means of maintaining colonization vitality [25]. Therefore, the symbiosis between soybean and *P. indica* under three media was explored (Supplementary Figure S1B). After 15 days of co-cultivation, there was no significant difference in the soybean growth phenotype between PNM and Hoagland media, while the growth of soybeans in 1/2 MS medium was significantly inhibited, so 1/2 MS medium was not suitable as a symbiotic medium.

**Figure 1:**
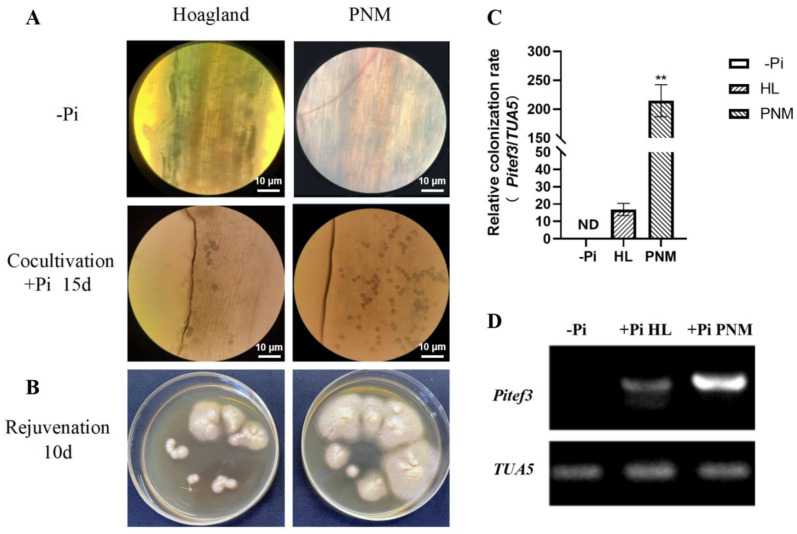
Differences in the colonization of *Piriformis indica* in PNM and Hoagland media. (**A**) typical trypan blue detection result of *P. indica*-colonization in soybean root (light microscope, magnification 200× scale, the scale bar indicates 10 μm). (**B**) The rejuvenation of symbiotic *P. indica* (colonized roots from PNM or Hoagland medium grown for 10 days in PDA media). (**C**) Molecular identification of the marker gene *Pitef1* in *P. indica*-colonized soybean lateral roots between PNM or Hoagland (HL) media by qRT-PCR and (**D**) RT-PCR. ND: non-detection. +Pi: *P. indica*-colonized. −Pi: non-colonized plants. *GmTAU5* was an internal reference gene. The values are the means ± SDs of three replicates; ** indicates an extremely significant difference (*p* < 0.001).

Although there was no difference in the growth of *P. indica* and soybean seedlings in PNM and Hoagland media, trypan blue staining showed that there was a great difference in the colonization of *P. indica* in soybean lateral roots (Figure 1). After 15 days of symbiotic culture, the number of characteristic blue-purple chlamydospores detected in lateral soybean roots in the PNM symbiotic medium was significantly higher than that in the Hoagland symbiotic medium (Figure 1A). The expression of the *PiTef3* gene in soybean lateral roots was detected by qPCR and RT-PCR (Figure 1C,D). The expression level of the *PiTef3* gene in the roots in the PNM symbiotic medium was remarkably higher than that in Hoagland (HL) symbiotic medium (*p* < 0.001). In addition, regarding the rejuvenation rate of symbiotic *P. indica*, the soybean roots in the PNM symbiotic medium were faster than that in HL symbiotic medium (Figure 1B). It can be seen that the PNM medium was the best symbiotic medium for *P. indica* and soybean.

**Figure 2:**
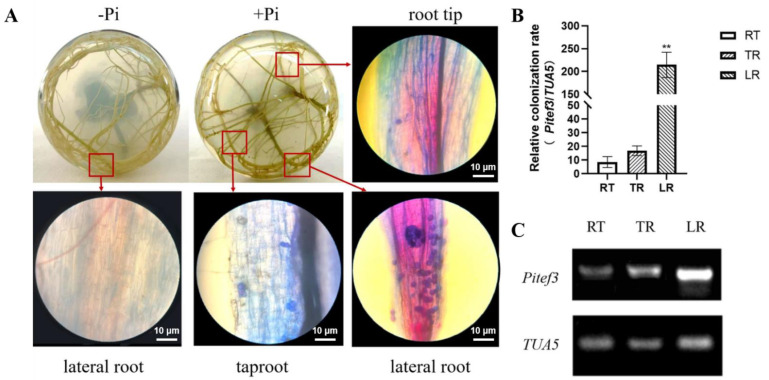
Differences in the colonization and distribution of *Piriformis indica* in the lateral roots, taproots, and root tips of soybean. (**A**) Typical trypan blue detection result of *P. indica*-colonization in soybean roots (light microscope, magnification 200× scale, the scale bar indicates 10 μm). (**B**) The expression of the *Pitef1* gene in *P. indica*-colonized soybean lateral roots (LR), taproots (TR), and root tips (RT) by qRT-PCR and (**C**) RT-PCR. ND: non-detection. +Pi: *P. indica*-colonized. −Pi: non-colonized plants. *GmTAU5* was an internal reference gene. The values are the means ± SDs of three replicates; ** indicates an extremely significant difference (*p* < 0.01).

Studies have shown that *P. indica* colonization in plant roots has a certain specificity [23]. The distribution of *P. indica* colonization in soybean roots was analyzed (Figure 2). It could be seen by trypan blue staining that blue-purple chlamydospores could be seen in root tips, main roots, and lateral roots (Figure 2A), but the numbers were significantly different. The results of qPCR and RT-PCR (Figure 2B) showed that the expression level of the *PiTef3* gene was higher in lateral roots than that in taproots and root tips (*p* < 0.001), and the expression level of the *PiTef3* gene in root tips was the lowest. Therefore, lateral roots should be selected for cultivation in rejuvenation.

### 2.2. Piriformis indica Improves the Salt Tolerance of Soybean

**Figure 3:**
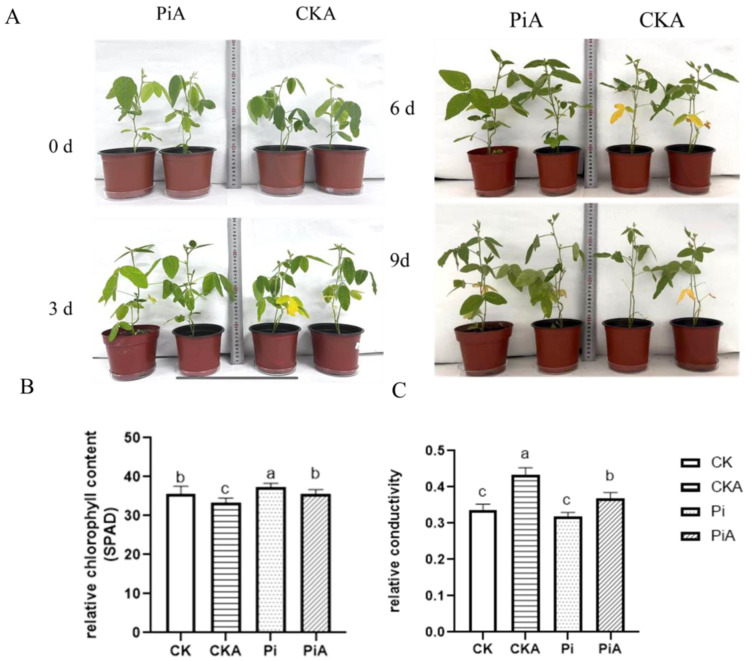
Effect of *P. indica* on the growth of the soybean (**A**), relative chlorophyll content (**B**), and relative conductivity (**C**) before and after salt treatment for nine days. CK: *P. indica* non-colonized control plants; Pi: plants inoculated with *P. indica*. CKA: Control plants were treated with 200 mmol/L NaCl. PiA: inoculated soybeans were treated with 200 mmol/L NaCl. Different letters above the bars indicate a significant difference (*p* < 0.05) from CK (0 h) between the CK and Pi groups. Error bars represent SDs (n = 3).

When the symbiotic system was established for 15 days, 200 mmol/L NaCl salt stress was used to compare the differences in the phenotypes of inoculated and uninoculated plants under salt stress (Figure 3A). With the increase of salt stress time, soybean leaves in the control group (CKA group) gradually wilted and turned yellow and even fell off after nine days of salt stress, while the inoculated soybean (PiA group) showed a small amount of leaf wilting and yellowing without any leaf shedding. Chlorophyll content and relative electrical conductivity in each group of samples were measured under salt stress for nine days (Figure 3B,C). Before salt stress treatment, the chlorophyll (Chl) content of inoculated soybeans (Pi) was significantly higher than that of the control (CK) (*p* < 0.05). After salt stress treatment, in soybean leaves, Chl decreased sharply, but in the PiA group, it was significantly higher than that in the CKA group. Similarly, after the salt stress treatment, the relative conductivity in the CKA group was significantly higher than that in the PiA group. Thus, salt stress caused salt damage to soybean, and the colonization of *P. indica* could alleviate the damage of salt stress.

### 2.3. Piriformis indica Reduces Oxidative Damage of Soybean under Salt Stress

**Figure 4:**
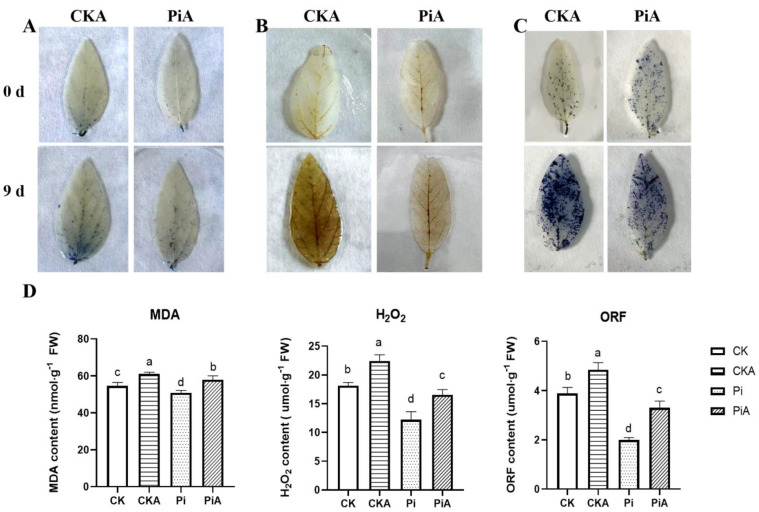
Oxidative damage of soybean inoculated with *Piriformis indica* (Pi) and control (CK) after salt stress. (**A**) Trypan blue staining, (**B**) DAB (3,3′-Diaminobenzidine), and (**C**) NBT (Nitrotetrazolium blue chloride). (**D**) The contents of malondialdehyde (MDA), H_2_O_2_, and O_2_^−^ (oxygen free radical, ORF). CK: *P. indica* non-colonized control plants; Pi: plants inoculated with *P. indica*. CKA: control plants were treated with 200 mmol/L NaCl. PiA: inoculated soybeans were treated with 200 mmol/L NaCl. Different letters above the bars indicate a significant difference (*p* < 0.05). Error bars represent SDs (n = 9).

The damage degree of soybean leaves after salt stress and the content of hydrogen peroxide (H_2_O_2_) and superoxide anion (O_2_^−^) was evaluated by trypan blue staining, DAB (3,3′-diaminobenzidine), and NBT (nitrotetrazolium blue chloride), and the contents of MDA, H_2_O_2_, and O_2_^−^ were determined correspondingly (Figure 4). The results of trypan blue staining showed that after nine days of salt stress, the number of locus coeruleus in the PiA group was not significantly different from that in the unstressed treatment, while that in the CKA group increased significantly. The change in MDA content was consistent with it; the MDA content of the PiA group was lower than that of the CKA group CK (*p* < 0.05). The changes of DAB and NBT staining and H_2_O_2_ and O_2_^−^ content corresponded and were consistent with the results. After salt stress, the CKA leaves had darker brown spots and more blue spots. Meanwhile, the CKA group had higher H_2_O_2_ and O_2_^−^ content than the PiA group. It is worth mentioning that the contents of MDA, H_2_O_2_, and O_2_^−^ in the Pi group were significantly lower than those in the CK group when untreated.

**Figure 5:**
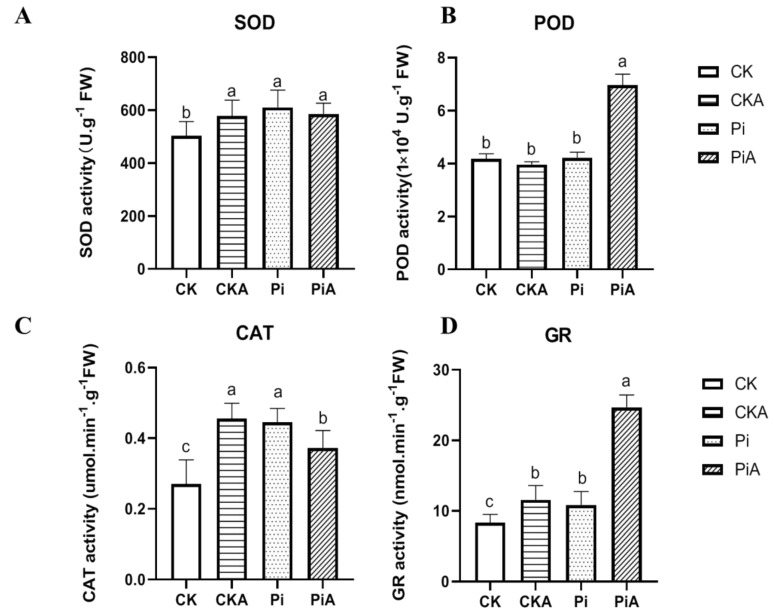
Effects of *Piriformis indica* on antioxidant enzyme activities in leaves of soybean under salt stress. The enzyme activities of SOD (**A**), POD (**B**), CAT (**C**), and GR (**D**) were detected in the leaves of non-colonized plants (CK) compared to those colonized with *P. indica* (Pi). CKA: control plants were treated with 200 mmol/L NaCl. PiA: inoculated soybeans were treated with 200 mmol/L NaCl. The error bars indicate the standard deviation (n = 9). The different letters above the bars indicate a significant difference (*p* < 0.05).

The antioxidant enzyme system is an important barrier for plant cells to avoid stress, and the reduction of ROS accumulation is closely related to the enhancement of antioxidant enzyme activity [26]. The enzyme activities of SOD, POD, CAT, and GR were measured before and after salt stress (Figure 5) to evaluate the effect of *P. indica* inoculation on the soybean antioxidant enzyme system under salt stress. The SOD activity of soybeans inoculated with *P. indica* was significantly higher than that in the non-inoculated group (Figure 5A) without salt treatment. Similarly, the enzyme activity of CAT in the Pi group was significantly higher than that in the CK group (*p* < 0.01), which was 1.8 times that of the CK group. After salt stress, the CAT enzyme activity in leaves of the PiA group decreased, while that of the CKA group increased, and the CAT enzyme activity in the CKA group was significantly higher than that in the PiA group (Figure 5C). While there was no difference between the CK and Pi groups, after salt stress, the POD enzyme activity in the PiA group increased dramatically, which was 1.79 times that of the CKA group (Figure 5B). Similar to SOD and CAT, the GR enzyme activity in the Pi group was significantly higher than that in the CK group under the same conditions. After salt stress treatment, the GR enzyme activity was significantly increased, and in the PiA group, it was 2.2 times higher than in the CKA group.

### 2.4. Effects of Piriformis indica on Na^+^ and K^+^ Ion Concentrations in Soybean Roots and Leaves under Salt Stress

**Figure 6:**
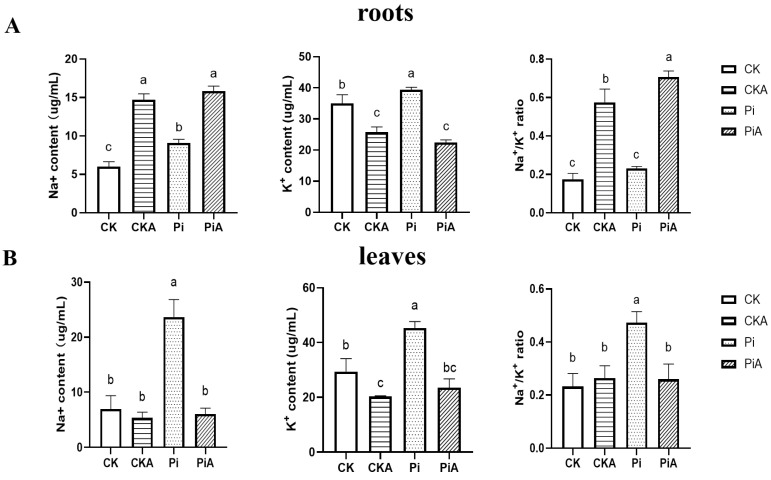
Changes in Na^+^ and K^+^ contents and Na^+^/K^+^ ratio in soybean roots (**A**) and leaves (**B**) of *P. indica* colonization (Pi) and non-colonized plants (CK) after salt treatment for nine days. The values are the means ± SDs of three replicates. Different letters above the bars indicate a significant difference (*p* < 0.05).

Studies have shown that *P. indica* can improve the absorption of ions in symbiotic plants and can act as a counter ion under salt stress to reduce salt toxicity [10]. Under non-salt conditions, the content of Na^+^ and K^+^ in the soybean roots inoculated with *P. indica* was significantly low and higher than that of the control group (*p* < 0.05), respectively (Figure 6). There was no significant difference in the Na^+^/K^+^ ratio between the two groups. After salt stress, the Na^+^ content in roots increased, and the K^+^ content decreased. Although there was no difference between the two groups, there was a difference in the Na^+^/K^+^ ratio, and the PiA group was significantly high than the CKA group. Under normal conditions, the contents of Na and K ions in the soybean leaves of symbiotic *P. indica* were higher than those in the CK group by 3.33 and 1.25 times, respectively, and the Na^+^/K^+^ ratio was also higher than that in the control group. After salt stress, in the CKA group, only K^+^ decreased slightly, while in the PiA group, Na and K ions and the Na^+^/K^+^ ratio decreased significantly.

### 2.5. Effects of Inoculation of Piriformis indica on the Expression of Ion Transport Regulated Genes in Soybean Roots under Salt Stress

**Figure 7:**
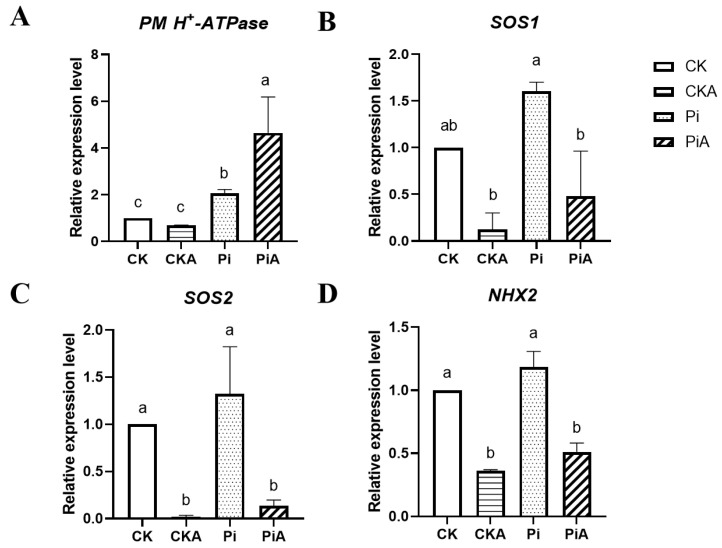
The relative gene expression levels of (**A**) *PM H^+^-ATPase*, (**B**) *SOS1*, (**C**) *SOS2*, and (**D**) *NHX2* gene from *P. indica*-colonized (Pi) and non-colonized (CK) soybean roots under salt stress. Values represent means ± standard deviation of three replicates. Different lowercase letters above the bars indicate statistically significant differences at the 0.05 level by Duncan’s multiple-range test.

Under salt stress, the ion balance and transport were closely related to the expression of *PM H^+^-ATPase*, *SOS1*, *SOS2*, and *NHX*-related genes. Therefore, the expression changes of ion-regulated genes in symbiotic soybean roots of *P. indica* before and after salt stress were determined (Figure 7). Except for the *NHX2* gene, there were certain differences between the two groups. In the CK group, the expression of the *PM H^+^-ATPase* gene did not change significantly after salt stress, but it was significantly up-regulated in the PiA group. Similarly, the expression of the *SOS1* gene in the PiA group was significantly higher than that in the CKA group, which was 3.85 times that of the CKA group. The *SOS2* gene was significantly decreased under salt stress. The expression of the *SOS2* gene in the Pi and PiA groups was significantly higher than that in the CK and CKA groups. It can be seen that the symbiosis of *P. indica* enhances the expression of soybean *PM H^+^-ATPase*, *SOS1*, and *SOS2* genes.

## 3. Discussion

Even though *Piriformospora indica* has been reported to promote plant biomass production in many plant systems [27], and it also has been shown that *P. indica* can interact with more roots than 30 plant families [15,28,29], the colonization efficiency and symbiosis of *P. indica* functionality are highly dependent on specific plant hosts and symbiotic conditions [11,30]. There are few reports on the symbiosis between *P. indica* and soybean, so it is necessary to establish an efficient symbiotic system for the application of *P. indica* in promoting soybean yield. In this study, the PNM medium was determined to be the most suitable medium for the in vitro co-culture of soybean and *P. indica*. The colonization efficiency and rejuvenation speed of fungus on the PNM medium were better than those in the Hoagland and 1/2 MS media, which are only suitable for plant growth. The results showed that the colonization of *P. indica* in soybean roots was selective, and the colonization rate of lateral roots was the highest, which was better than that of main roots and root tips. This result is similar to the colonization distribution of *P. indica* in other plants [23].

That *P. indica* improves host abiotic stress has been verified in tomato [31,32], Arabidopsis [13,33], rice [15], banana [16,28], barley [10,34], and other plants [35]. However, the exact mechanism by which *P. indica* promotes plant growth is specific to the different host plants, and the symbiotic system of *P. indica* and soybean and its salt tolerance function is rarely studied. In this study, the salinity tolerance of soybeans inoculated with *P. indica* was significantly improved. The main changes were that the chlorophyll content in leaves increased, the relative conductivity decreased, the activity of antioxidant enzymes increased, the accumulation of reactive oxygen species (ROS) decreased, the contents of Na^+^ and K^+^ ions in roots and leaves changed, and ion-balance related genes upregulated, etc. These results share similarities and differences with its performance in other *P. indica*-inoculated plants.

Wu et al. found that *P. indica* improved gerbera growth by increasing chlorophyll content and photosynthetic characteristics [27]. Dan Li et al. also found that *P. indica* colonization increased the photochemical conversion efficiency and electron transport rate of banana leaves under cold treatment [16]. The colonization of *P. indica* in *A. thaliana* under salt stress led to an increase in the effective transfer of electron flow in PS II, which alleviated damage by salt stress to plants [36,37]. In the present study, the relative content of chlorophyll in soybean inoculated with *P. indica* was higher than that in non-inoculated soybean. After salt stress, the relative content of chlorophyll decreased, but it was also significantly higher than that in non-inoculated soybean. Correspondingly, the inoculated soybean was more luxuriant than the non-inoculated soybean, while the non-inoculated soybean leaves turned yellow and fell off under salt stress. That is, *P. indica* could promote soybean growth and salt tolerance by increasing chlorophyll content and enhancing its photosynthesis and biomass.

It is essential for plants to maintain a balance between generating and scavenging ROS in order to be adaptive to biotic and abiotic stress [38]. Stress-induced oxidative damage is indicated by MDA content. In this study, soybean plants colonized by *P. indica* exhibited lower contents of MDA under salt treatment than non-colonized plants, indicating that ROS-mediated lipid peroxidation was mitigated within the symbiosis (Figure 4B). There was a potential link between the increase in MDA levels and the accumulation of H_2_O_2_ and O_2_^−^ in plants subjected to high salinity (Figure 4C,D). The levels of H_2_O_2_ and O_2_^−^ in the non-colonized plants were 1.44 and 1.56 times greater than those of colonized plants, respectively. It is worth mentioning that without salt treatment, the contents of MDA, H_2_O_2_, and O_2_^−^ in soybeans colonized by *P. indica* were all significantly lower than those in the uncolonized plants (*p* < 0.01 or *p* < 0.05). Thus, it was shown that the antioxidant enzyme system was induced and enhanced by *P. indica* treatment. This result was consistent with previous studies [39,40].

Generally, an increased capacity of antioxidant enzymes in salt-exposed plants is one of the reasons for salt stress tolerance. It has been proposed that salt tolerance might be enhanced through the activation of ROS-scavenging enzymes by *P. indica* colonization [17]. Our results were consistent with those obtained by Khalid M. et al. There was a significant increase in SOD, CAT, and GR enzyme activities in soybean colonized with *P. indica* compared to non-colonized plants under the same conditions. As a result of salt treatment, the POD and GR enzyme activities were significantly up-regulated in the colonized soybean, which was significantly higher than that in the uncolonized soybean (Figure 5). It seems that the significantly lower H_2_O_2_ and O_2_^−^ content in the colonized soybeans could be attributed to enhanced antioxidant enzyme activity after salt treatment. CAT is a major H_2_O_2_-scavenging enzyme; it can scavenge H_2_O_2_ into O_2_ and H_2_O [41]. The CAT activity in the Pi group noticeably increased by 184.6% compared with the CK group. Correspondingly, H_2_O_2_ concentration decreased significantly as CAT activity increased. Similarly, SOD enzymes can scavenge superoxide anions, and SOD enzyme activity was negatively correlated with O_2_^−^. After salt stress, the enzymatic activity of CAT and SOD did not change significantly due to low levels of H_2_O_2_ and O_2_^−^. GR is one of the key enzymes in the glutathione redox cycle and participates in the AsA-GSH cycle pathway. It catalyzes NADPH to reduce GSSG to regenerate GSH [42]. GR enzyme activity was significantly increased in the PiA group after salt stress. The increase in the enzyme activity in the colonized soybeans also played a positive role in scavenging other ROS. Although the change in POD enzyme activity was different from that of the other three enzymes, POD enzyme activity also increased significantly under salt stress in the colonized soybeans. All these indicated that *P. indica* increases CAT, SOD, GR, and POD activity and participates in the elimination of excess free radicals, accelerates the scavenging efficiency of H_2_O_2_ and O_2_^−^ and other ROS, and mitigates the damage of the cell membrane structure caused by salt stress.

In addition, some researchers believed that *P. indica* was capable of activating the antioxidant function in host plants, but the antioxidant enzyme’s responses are host-specific [15,29]. Chen et al. also found *P. indica* co-culture significantly increased salt tolerance of *Gerbera jamesonii* with low levels of MDA and hydrogen peroxide, whereas the activities of APX, CAT, POD, GR, and SOD and the levels of AsA and GSH did not increase significantly in *P. indica* colonization under salt stress [43]. Li et al. analyzed the activities of four antioxidant enzymes (APX, CAT, POD, and SOD) in the leaves and roots of sweet potatoes. Only the CAT activity was significantly enhanced in whole plants after *P. indica* colonization [44]. In this study, unexpectedly, although *P. indica* colonization conferred greater tolerance to salt stress, the activities of SOD and CAT did not increase significantly after salt treatment. Therefore, the mechanism of detoxification induced by *P. indica* may differ in different plants and may not be regulated by enzymatic and non-enzymatic ROS scavengers in certain plants.

As a result of salt stress, the root zone is up-regulated in K^+^ efflux [45], and Na^+^ toxicity is increased, decreasing K^+^ uptake and reducing cell expansion, stomatal opening, and photosynthesis rates [46]. In this study, it was found that inoculated and uninoculated soybean roots and leaves did not differ significantly in the content of Na^+^, K^+^ ion, and Na^+^/K^+^ ratio after salt treatment. However, it is worth mentioning that compared with the uninoculated soybean, Na^+^ ion content in the inoculated soybean roots decreased significantly, and K^+^ ion content increased significantly, while in the leaves, Na^+^ and K^+^ ions increased significantly, and the Na^+^/K^+^ ratio significantly increased. The results are in contrast with Abdelaziz et al. [19], who found that the Na^+^/K^+^ ratios in shoots and roots of colonized plants were lower than in non-colonized plants due to the higher K^+^ concentration and increasing transcripts level of *LeNHX1* (Na^+^/H^+^ exchanger) gene observed in leaves and roots of colonized plants under saline treatment. In contrast, the expression of the *GmNHX2* gene was not significantly different between inoculated and uninoculated soybean roots (Figure 7). It could be seen that the improvement in soybean salt tolerance by the colonization of *P. indica* was not only reflected in the regulation of ion homeostasis but also may be related to the transport of ions.

It is important for plants to regulate sodium/potassium transporter genes when faced with salt stress. In this respect, Abdelaziz et al. [33] found that *P. indica* enhanced the Na^+^/K^+^ ratio in Arabidopsis by regulating *HKT1*, *KAT1*, and *KAT2*. Abdelaziz et al. and Ghorbani et al. [47] also found that salt stress increased the transcript levels of the *NHX1*, *NHX2*, *NHX3*, *NHX4*, and *SOS1* (*NHX*7) genes in tomato leaves colonized by *P. indica*. In soybean, *SOS1* and *SOS2* were induced by *P. indica* colonization, but the expressions of the *NHX2* gene did not significantly differ in colonized and non-colonized soybean roots. In plants, the salt overly sensitive (SOS) pathways expel Na^+^ from the cytoplasm [48]. SOS1, SOS2, and SOS3 are the main members of the SOS pathway [49]. SOS2 phosphorylates and activates a Na^+^/H^+^ antiporter (SOS1), resulting in extensive Na^+^ exclusion from the cytoplasm [50]. Consequently, the induction of *SOS1* and *SOS2* by *P. indica* colonization suggests its role in the exclusion of Na^+^ in colonized soybean roots under salt stress. Abdelaziz et al. tested the expression levels of the intracellular antiporter *LeNHXs1-4* genes under salt stress, but only *LeNHX1* was increased in the transcripts in leaves of colonized plants [19]. However, *P. indica* colonization positively regulated the transcription level of *NHX2* and *SOS1* in gerbera seedlings, except for *NHX1* and *NHX4* genes [47]. Consequently, the regulation of NHX proteins in soybean by *P. indica* requires further exploration. To address whether *P. indica* colonization may respond to salt stress by regulating other ion channels, the expression of *PM H^+^-ATPase* in roots was tested. Unlike *SOS1* and *SOS2* genes, under normal conditions, *P. indica* colonization did not change the transcription level of the *PM-H^+^-ATPase* gene, but after salt stress, the *PM-H^+^-ATPase* gene was significantly up-regulated compared with that in uncolonized soybean. Several studies have shown that H^+^-ATPase can maintain the intracellular pH level under alkaline stress [51,52,53]. *PM-H^+^-ATPase* is a key regulator of NaCl tolerance as it provides a proton-driving force for Na^+^/H^+^ exchange [54]. The effect of *P. indica* colonization on the transcription of the *PM-H^+^-ATPase* gene has not been reported yet, and its regulatory mechanism needs to be further studied.

Although we found that the colonization of *P. indica* in soybean root can improve the salt tolerance of soybean, however, there are still some problems to be solved; for example, does the degree of salt tolerance for soybean depend on the concentration of *P. indica*? Does *P. indica* interfere with the growth and development of nitrogen-fixing bacteria resulting in the low ability of nitrogen-fixing in soybean? How long can the salt tolerance ability be maintained in *P. indica*-colonized plants? After this, we will focus on the application of *P. indica*.

In conclusion, this study established the symbiotic system between *P. indica* and soybean plants. *P. indica* colonization promotes soybean growth by increasing the content of chlorophyll and alleviates the negative impacts of salinity stress by increasing antioxidant activity and scavenging for ROS by upregulating the expression of *PM-H^+^-ATPase*, *SOS1*, and *SOS2* genes. These improvements in salt tolerance, mediated by *P. indica*, offer a promising strategy for the agricultural production of soybean plants in saline-alkali soils.

## 4. Materials and Methods

### 4.1. Symbiotic Culture of Piriformis indica and Soybean in Different Media

*Piriformis indica* (strain DSM11827) was acquired from Professor Chu Wu from the College of Horticulture and Gardening, Yangtze University (Jinzhou, China). *P. indica* was cultured on PDA (potato dextrose agar) medium at 30 °C for 2 weeks in the dark. High-quality soybean seeds (‘Dongnong 50’, College of Life Science, Northeast Agricultural University, Harbin, China) were screened and sterilized by chlorine fumigation. About 1 cm³ of activated mycelia was cut from the PDA medium and placed in the middle of flasks containing each of the three media (Hoagland, PNM, and ½ MS, Supplementary Table S1) [55]. In culture flasks, sterilized soybean seeds were placed about 2 cm around the mycelium.

### 4.2. Detection of the Colonization Efficiency and Distribution of P. indica in the Soybean Roots

After *P. indica* inoculation for 14 days, soybean roots were randomly selected, washed thoroughly with sterile water, and then the main root, lateral root, and root tip were cut into 1 cm segments for trypan blue staining [30].

At the same time, the colonization efficiency and distribution of *P. indica* in three symbiotic media were evaluated by the expression difference of the *Pitef1* gene [44]. Pitef1 gene expression changes were monitored by real-time qPCR and RT-PCR. Soybean seedlings were randomly taken out from the PNM and Hoagland medium inoculated with *P. indica*, and the main roots, lateral roots, and root tips were taken. Total RNA was extracted by Ultrapure RNA kit (Kangwei Century Biotechnology Co., Ltd., Beijing, China). cDNA was reverse-transcribed, and the quality of the extracted cDNA was verified via PCR using *TUA5* from soybean as an internal reference gene. The primer sequences of *TUA5* and *Pitef* are shown in Supplementary Table S2. The relative expression fold change of the target gene was calculated using the 2^−∆∆Ct^ method. Three biological replicates and three technical replicates were performed for each root.

### 4.3. Rejuvenation of P. indica

The lateral soybean roots were taken out from the PNM and Hoagland and rinsed with sterile water 3–4 times. The roots were then cut into 1 cm lengths, placed into the prepared PDA solid medium, and stored at 30 °C in an incubator for 10 days.

### 4.4. P. indica Inoculation and Salt Treatment

Germinated soybean seeds were transplanted into plastic pots containing nutrient soil. After growing for 2 weeks at 25 °C, a photoperiod of 16/8 h (day/night, 1500 ± 200 lx), and 60–80% relative humidity, seedlings at the five- to six-leaf stage were used for *P. indica* inoculation.

For plant inoculation, 250 mL of liquid ASP medium (see Supplementary Table S1) was supplied by five fungal plugs after rejuvenation and incubated for 15 days at 24 °C and 120 rpm on a rotary shaker. Pure white mycelium was washed three times with sterile ddH_2_O. An amount of 2 g of *P. indica* mycelium was mixed with 100 mL sterilized ddH_2_O to *P. indica* suspension. A total of 10mL of suspension was inoculated into the roots of soybean plants, using ddH_2_O without *P. indica* as the non-inoculated control [19].

The salt treatment was initiated two weeks after plant inoculation by watering with 200 mmol/L NaCl. The control was irrigated water. The shoot and root samples from *P. indica*-inoculated and non-inoculated plants under normal and salt treatment were harvested 9 days after salt treatment. Three plants per pot (as one replication) were harvested from three pots (as biological replicates) and used for the physiological analysis. The collected samples for RNA extraction were immediately frozen in liquid N2.

### 4.5. Determination of Soybean Physiological and Biochemical Indicators under Salt Stress

The determination of physiological and biochemical indicators is divided into two parts. First, the relative chlorophyll content (Chl) using the SPAD chlorophyll meter. Each recorded value included three biological replicates and five technical replicates. The relative conductivity of the leaves was then determined by the vacuum method. After that, the malondialdehyde (MDA) content was measured by UV-vis spectrophotometer and Solarbio kit (Beijing Solarbio Technology Co., Ltd., Beijing, China). The MDA content was calculated by subtracting the absorbance at 532 and 600 nm. Second, the activities of the antioxidant enzymes were measured in wild-type and transgenic plants after salt stress. The content of hydrogen peroxide (H_2_O_2)_ and superoxide anion (OFR), as well as superoxide dismutase (SOD) activity, catalase (CAT), peroxidase (POD) activity, and glutathione reductase (GR) activity were measured. Every value included three biological replicates and three technical replicates. The intact plant leaves of *P. indica* and the control group soybean were taken before the salt stress treatment and on the ninth day after the treatment, respectively, and the plant materials were placed in DAB, NBT, and trypan blue staining solution. They were placed in a vacuum for 30 min and in the stain overnight. The next day, the leaves in DAB and NBT were transferred to a decolorizing solution (ethanol: acetic acid: glycerol = 3:1:1) in a boiling water bath to decolorize until there was no chlorophyll residue. The leaves in the trypan blue staining solution were decolorized in 40% chloral hydrate for 24 h, and the staining results were observed.

### 4.6. Analysis of Na^+^ and K^+^ Ions in Soybean

The control and *P. indica* inoculated soybean roots and leaves were dried to stable weight at 80 °C. A certain amount of nitrification solution was added, and 0.05 g of the material was ground into dry powder, extracted, and filtered in a 90 °C water bath and measured by flame atomic absorption spectrometry. Amounts of 0, 2, 5, 10, 20, and 40 mL of the mixed standard solution were pipetted into a 50 mL volumetric flask, and equal amounts of K and Na standards were added. The nitrification solution was then diluted with deionized water to obtain mixed standard solutions containing K at 0, 2, 5, 10, 20, and 40 μg·mL^−1^ and Na at 0, 5, 12.5, 25, 50, 100 μg·mL^−1^. For each sample and time point, three biological replicates and three technical replicates were performed after making a standard curve with the prepared mixed standard solutions [53].

### 4.7. Differential Expressions of Ion Transporter Genes in P. indica Inoculated Roots under Salt Stress

In order to further explore the effect of inoculation of *P. indica* on ion balance under salt stress, the expression of *PM H^+^-ATPase*, *SOS1*, *SOS2*, and *NHX1* (the primer sequences are shown in Supplementary Table S2) in soybean roots was detected by the real-time PCR method. *TUA5* was used as the internal reference gene. The relative expression fold change of the target gene was calculated using the 2^−∆∆Ct^ method. Three biological replicates and three technical replicates were performed. According to the previously established protocol, RNA quality analysis and primer specificity testing were carried out [53,56].

### 4.8. Statistical Analysis

All data were organized using Microsoft Excel, 2010; GraphPad Prism version 9.0.0 (www.graphpad.com/updates/prism-900-release-notes, accessed on 28 October 2020) was used to plot the data; one-way ANOVA and Duncan’s multiple-range test were used to analyze the data.

## Supplementary Materials

The following supporting information can be downloaded at: https://www.mdpi.com/article/10.3390/ijms232314961/s1.

## References

[1] Litalien A., Zeeb B. (2020). Curing the earth: A review of anthropogenic soil salinization and plant-based strategies for sustainable mitigation. Sci. Total Environ..

[2] Khalid M., Saeed ur R., Huang D.F. (2019). Molecular mechanism underlying *Piriformospora indica*-mediated plant improvement/protection for sustainable agriculture. Acta Biochim. Biophys..

[3] De Vries F.T., Grifths R., Knight C.G., Nicolitch O., Williams A. (2020). Harnessing rhizosphere microbiomes for drought-resilient crop production. Science.

[4] De la Fuente C.C., Simonin M., King E., Moulin L., Bennett M.J., Castrillo G., Laplaze L. (2022). An extended root phenotype: The rhizosphere, its formation and impacts on plant ftness. Plant J..

[5] Mengistu A.A. (2020). Endophytes: Colonization, behaviour, and their role in defense mechanism. Int. J. Microbiol..

[6] Peškan-Berghöfer T., Shahollari B., Giong P.H., Hehl S., Markert C., Blanke V., Kost G., Varma A., Oelmüller R. (2004). Association of *Piriformospora indica* with *Arabidopsis thaliana* roots represents a novel system to study beneficial plant-microbe interactions and involves early plant protein modifications in the endoplasmic reticulum and at the plasma membrane. Physiol. Plant.

[7] Kumari R., Kishan H., Bhoon Y.K., Varma A. (2003). Colonization of cruciferous plants by *Piriformospora indica*. Curr. Sci..

[8] Qiang X., Weiss M., Kogel K.H., Schäfer P. (2012). *Piriformospora indica*—A mutualistic basidiomycete with an exceptionally large plant host range. Mol. Plant Pathol..

[9] Bagheri A.A., Saadatmand S., Niknam V., Nejadsatari T., Babaeizad V. (2014). Effects of *Piriformospora indica* on biochemical parameters of *Oryza sativa* under salt stress. Int. J. Biosci..

[10] Baltruschat H., Fodor J., Harrach B.D., Niemczyk E., Barna B., Gullner G., Janeczko A., Kogel K.H., Schäfer P., Schwarczinger I. (2008). Salt tolerance of barley induced by the root endophyte *Piriformospora indica* is associated with a strong increase in antioxidants. New Phytol..

[11] Mensah R.A., Li D., Liu F., Tian N., Sun X., Hao X., Lai Z., Cheng C. (2020). Versatile *Piriformospora indica* and its potential applications in horticultural crops. Hort. Plant J..

[12] Hui F., Liu J., Gao Q., Lou B. (2015). *Piriformospora indica* confers cadmium tolerance in *Nicotiana tabacum*. J. Environ. Sci..

[13] Jiang W., Pan R., Wu C., Xu L., Abdelaziz M.E., Oelmüller R., Zhang W. (2020). *Piriformospora indica* enhances freezing tolerance and post-thaw recovery in Arabidopsis by stimulating the expression of *CBF* genes. Plant Signal Behav..

[14] Rahman S.U., Khalid M., Kayani S.I., Tang K. (2020). The ameliorative efects of exogenous inoculation of *Piriformospora indica* on molecular, biochemical and physiological parameters of *Artemisia annua* L. under arsenic stress condition. Ecotoxicol. Environ. Saf..

[15] Tsai H.J., Shao K.H., Chan M.T., Cheng C.P., Yeh K.W., Oelmüller R., Wang S.J. (2020). *Piriformospora indica* symbiosis improves water stress tolerance of rice through regulating stomata behavior and ROS scavenging systems. Plant Signal Behav..

[16] Li D., Bodjrenou D.M., Zhang S., Wang B., Pan H., Yeh K.-W., Lai Z., Cheng C. (2021). The Endophytic Fungus *Piriformospora indica* Reprograms Banana to Cold Resistance. Int. J. Mol. Sci..

[17] Khalid M., Hassani D., Liao J., Xiong X., Bilal M., Huang D. (2018). An endosymbiont *Piriformospora indica* reduces adverse efects of salinity by regulating cation transporter genes, phytohormones, and antioxidants in *Brassica campestris* ssp. Chinensis. Environ. Exp. Bot..

[18] Munns R., Tester M. (2008). Mechanisms of Salinity Tolerance. Annu. Rev. Plant Biol..

[19] Abdelaziz M.E., Abdelsattar M., Abdeldaym E.A., Atia M.A.M., Mahmoud A.W.M., Saad M.M., Hirt H. (2019). *Piriformospora indica* alters Na^+^/K^+^ homeostasis, antioxidant enzymes and *LeNHX1* expression of greenhouse tomato grown under salt stress. Sci. Hortic..

[20] Ullah A., Li M., Noor J., Tariq A., Liu Y., Shi L. (2019). Effects of salinity on photosynthetic traits, ion homeostasis and nitrogen metabolism in wild and cultivated soybean. PeerJ.

[21] Sun T.J., Ma N., Wang C.Q., Fan H.F., Wang M.X., Zhang J., Cao J.F., Wang D.M. (2021). A Golgi-Localized Sodium/Hydrogen Exchanger Positively Regulates Salt Tolerance by Maintaining Higher K^+^/Na^+^ Ratio in Soybean. Front. Plant Sci..

[22] Nigam B., Dubey R.S., Rathore D. (2022). Protective role of exogenously supplied salicylic acid and PGPB (*Stenotrophomonas* sp.) on spinach and soybean cultivars grown under salt stress. Sci. Hortic..

[23] Wang X.H., Chang W., Song F.Q. (2021). Roles of *Serendipita indica* in phytoremediation of heavy metal pollution. Sci. Sin. Vitae.

[24] Varma A., Singh A., Sudha, Sahay N.S., Sharma J., Roy A., Kumari M., Rana D., Thakran S., Deka D., Hock B. (2001). *Piriformospora indica*: An Axenically Culturable Mycorrhiza-Like Endosymbiotic Fungus. Fungal Associations. The Mycota, Volume 9.

[25] Johnson J.M., Sherameti I., Ludwig A., Nongbri P.L., Sun C., Lou B., Varma A., Oelmu¨ller R. (2011). Protocols for *Arabidopsis thaliana* and *Piriformospora indica* co-cultivation—A model system to study plant benefificial traits. Endocyt. Cell Res..

[26] CHullabaloo D., Analin B., Mohanan A., Bakka K. (2021). Differential modulation of photosynthesis, ros and antioxidant enzyme activities in stress-sensitive and -tolerant rice cultivars during salinity and drought upon restriction of cox and aox pathways of mitochondrial oxidative electron transport. J. Plant Physiol..

[27] Wu H., Wang B., Hao X., Zhang Y., Wang T., Lu Z., Lai Z., Cheng C. (2022). *Piriformospora indica* promotes the growth and enhances the root rot disease resistance of gerbera. Sci. Hortic..

[28] Li D., Mensah R.A., Liu F., Tian N., Qi Q., Yeh K., Xu X., Cheng C., Lai Z. (2019). Efects of *Piriformospora indica* on rooting and growth of tissue-cultured banana (*Musa acuminata* cv. Tianbaojiao) seedlings. Sci. Hortic..

[29] Lin H.F., Xiong J., Zhou H.M., Chen C.M., Lin F.Z., Xu X.M., Oelmüller R., Xu W.F., Yeh K.W. (2019). Growth promotion and disease resistance induced in Anthurium colonized by the benefcial root endophyte *Piriformospora indica*. BMC Plant Boil..

[30] Liu H., Senthilkumar R., Ma G., Zou Q., Zhu K., Shen X., Tian D., Hua M.S., Oelmüller R., Yeh K.W. (2019). *Piriformospora indica*-induced phytohormone changes and root colonization strategies are highly host-specifc. Plant Signal Behav..

[31] Azizi M., Fard E.M., Ghabooli M. (2021). *Piriformospora indica* affect drought tolerance by regulation of genes expression and some morphophysiological parameters in tomato (*Solanum lycopersicum* L.). Sci. Hortic..

[32] Ghorbani A., Razavi S.M., Omran V.O.G., Pirdashti H. (2018). *Piriformospora indica* inoculation alleviates the adverse effect of NaCl stress on growth, gas exchange and chlorophyll fluorescence in tomato (*Solanum lycopersicum* L.). Plant Biol..

[33] Abdelaziz M.E., Kim D., Ali S., Fedorof N.V., Al-Babili S. (2017). The endophytic fungus *Piriformospora indica* enhances *Arabidopsis thaliana* growth and modulates Na^+^/K^+^ homeostasis under salt stress conditions. Int. J. Exp. Plant Biol..

[34] Murphy B.R., Doohan F.M., Hodkinson T.R. (2014). Yield increase induced by the fungal root endophyte *Piriformospora indica* in barley grown at low temperature is nutrient limited. Symbiosis.

[35] Xu L., Wu C., Oelmüller R., Zhang W. (2018). Role of Phytohormones in *Piriformospora indica*—Induced Growth Promotion and Stress Tolerance in Plants: More Questions Than Answers. Front. Microbiol..

[36] Johnson J.M., Alex T., Oelmüller R. (2014). *Piriformospora indica*: The versatile and multifunctional root endophytic fungus for enhanced yield and tolerance to biotic and abiotic stress in crop plants. J. Trop. Agric..

[37] Johnson J.M. (2014). The Role of Cytosolic Calcium Signaling in Benefificial and Pathogenic Interactions in *Arabidopsis thaliana*. Ph.D. Thesis.

[38] Nath M., Bhatt D., Prasad R., Gill S.S., Anjum N.A., Tuteja N. (2016). Reactive oxygen species generation-scavenging and signaling during plant-arbuscular mycorrhizal and *Piriformospora indica* interaction under stress condition. Front. Plant Sci..

[39] Nanda R., Agrawal V. (2018). *Piriformospora indica*, an excellent system for heavy metal sequestration and amelioration of oxidative stress and DNA damage in *Cassia angustifolia* Vahl under copper stress. Ecotoxicol. Environ. Saf..

[40] Alizadeh F.M., Pirdashti H., Yaghoubian Y., Babaeizad V. (2016). Effect of paclobutrazol and *Piriformospora indica* inoculation on antioxidant enzymes activity and morphological characteristics of green beans (*Phaseoluse vulgaris* L.) in chilling stress. J. Plant Process Funct..

[41] Veronica N., Subrahmanyam D., Kiran T.V., Yugandhar P., Bhadana V., Padma V., Jayasree G., Volet S. (2017). Infuence of low phosphorus concentration on leaf photosynthetic characteristics and antioxidant response of rice genotypes. Photosynthetica.

[42] Romero-Puertas M.C., Corpas F.J., Sandalio L.M., Marina L., Rodríguez-Serrano M., del Río L.A., Palma J.M. (2006). Glutathione reductase from pea leaves: Response to abiotic stress and characterization of the peroxisomal isozyme. New Phytol..

[43] Chen W.T., Lin F.Z., Lin K.H., Chen C.M., Xia C.S., Liao Q.L., Chen S.P., Kuo Y.W. (2022). Growth Promotion and Salt-Tolerance Improvement of Gerbera jamesonii by Root Colonization of *Piriformospora indica*. J. Plant Growth Regul..

[44] Li Q., Kuo Y.W., Lin K.H., Huang W.Q., Deng C.S., Yeh K.W., Chen S.P. (2021). *Piriformospora indica* colonization increases the growth, development, and herbivory resistance of sweet potato (*Ipomoea batatas* L.). Plant Cell Rep..

[45] Shabala S., Pottosin I. (2014). Regulation of potassium transport in plants under hostile conditions: Implications for abiotic and biotic stress tolerance. Physiol. Plant..

[46] Shabala S., Bose J., Fuglsang A.T., Pottosin I. (2016). On a quest for stress tolerance genes: Membrane transporters in sensing and adapting to hostile soils. J. Exp. Bot..

[47] Ghorbani A., Omran V.O.G., Razavi S.M., Pirdashti H., Ranjbar M. (2019). *Piriformospora indica* confers salinity tolerance on tomato (*Lycopersicon esculentum* Mill.) through amelioration of nutrient accumulation, K^+^/Na^+^ homeostasis and water status. Plant Cell Rep..

[48] Zhu J.-K. (2000). Genetic analysis of plant salt tolerance using Arabidopsis. Plant Physiol..

[49] Liu J., Ishitani M., Halfter U., Kim C.-S., Zhu J.-K. (2000). The *Arabidopsis thaliana SOS2* gene encodes a protein kinase that is required for salt tolerance. Proc. Natl. Acad. Sci. USA.

[50] Qiu Q.-S., Guo Y., Dietrich M.A., Schumaker K.S., Zhu J.-K. (2002). Regulation of SOS1, a plasma membrane Na^+^/H^+^ exchanger in *Arabidopsis thaliana*, by SOS2 and SOS3. Proc. Natl. Acad. Sci. USA.

[51] Heidi P.O., Ana G., Verena K., Evrim S., Huang S., Fang H., Lang V., Sydow K., Pöckl M., Schulze W.X. (2022). PH modulates interaction of 14-3-3 proteins with pollen plasma membrane H^+^-ATPases independently from phosphorylation. J. Exp. Bot..

[52] Zhou S., Wang P., Ding Y., Xie L., Li A. (2022). Modifification of plasma membrane H^+^-ATPase in Masson pine (*Pinus massoniana* Lamb.) seedling roots adapting to acid deposition. Tree Physiol..

[53] Zhang D., Zhang Z., Li C., Xing Y., Luo Y., Wang X., Li D., Ma Z., Cai H. (2022). Overexpression of *MsRCI2D* and *MsRCI2E* Enhances Salt Tolerance in Alfalfa (*Medicago sativa* L.) by Stabilizing Antioxidant Activity and Regulating Ion Homeostasis. Int. J. Mol. Sci..

[54] Gupta A., Shaw B.P., Roychoudhury A. (2021). NHX1, HKT, and monovalent cation transporters regulate K^+^ and Na^+^ transport during abiotic stress. Transporters and Plant Osmotic Stress.

[55] Kaefer E. (1977). Meiotic and mitotic recombination in Aspergillus and its chromosomal aberrations. Adv. Genet..

[56] Li C., Song T., Zhan L., Cong C., Xu H., Dong L., Cai H. (2021). Overexpression of *MsRCI2A, MsRCI2B*, and *MsRCI2C* in Alfalfa (*Medicago sativa* L.) Provides Different Extents of Enhanced Alkali and Salt Tolerance Due to Functional Specialization of MsRCI2s. Front. Plant Sci..

