# Supplementary material for: Symbiotic System Establishment between Piriformospora indica and Glycine max and Its Effects on the Antioxidant Activity and Ion-Transporter-Related Gene Expression in Soybean under Salt Stress
Source: Int J Mol Sci. 2022 Nov 29;23(23):14961. doi: 10.3390/ijms232314961 (PMC9739428; doi:10.3390/ijms232314961)
Supplement: Supplementary file 1 [file ijms-23-14961-s001.zip › ijms-1963786-supplementary.pdf]

Supplementary

Table S1. The composition of the culture medium.

Name	Element	Dosage （g/L）	pH
PDA	Peeled and sliced potato<br>Agar	200	
	Glucose	20	7
Aspergillus[55]	peptone	2	6.5
	Yeast extract	1	
	Casamino acid	1	
	Vitamins stock	1ml	
	1macro elements from stock	50ml	
	micro elements from stock	2.5ml	
	0.1mM $CaCl_2$	1ml	
	0.1mM $FeCl_3$	1ml	
1macro elements from stock	$NaNO_3$	120	
	KCl	10.4	
	$MgSO_4 \cdot 7H_2O$	10.4	
	KH_2PO_4	30.4	
micro elements from stock	$ZnSO_4 \cdot 7H_2O$	22	
	H_3BO_3	11	
	$MnCl_2 \cdot 4H_2O$	5	
	$FeSO_4 \cdot 7H_2O$	5	
	$CoCl_2 \cdot 6H_2O$	1.6	
	$CuSO_4 \cdot 5H_2O$	1.6	

	$(NH_4)_6Mo_7O_{27}\cdot 4H_2O$	1.1	
	Na_2EDTA	50	
Vitamins stock	Biotin	0.5	
	Nicotinamide	5	
	Pyridoxal phosphate	1	
	Amino benzoic acid	1	
	Riboflavin	2.5	
Modified PNM	KNO3	5.0 mM	
	$MgSO4\cdot 7H_2O$	2.0 mM	
	$Ca(NO_3)_2$	2.0 mM	
	1 Fe-EDTA/liter	2.5 ml	
	2 Micronutrient-mix/liter	1.0 ml	
	Agar (Serva)/liter	10.0 g	5.6
	• Sterilize at 121°C for 20 min		
	fifilter-sterilized 1M KH_2PO_4	2.5 ml	
1 Fe-EDTA			
	• Add 2.5 g $FeSO_4\cdot 7H_2O$ in 400 ml dH_2O		
	• Add 3.36 g $Na_2EDTA\cdot 2H_2O$		
	• Heat to boil in the microwave		
	• Stir for about 30 min while cooling		
	• Bring to the fifinal volume of 450 ml		
2 Micronutrient mix			
	H_3BO_3	70.0 mM	
	$MnCl_2\cdot 4H_2O$	14.0 mM	
	$CuSO_4\cdot 5H_2O$	0.5 mM	
	$ZnSO_4\cdot 7H_2O$	1.0 mM	
	$Na_2MoO_4\cdot 2H_2O$	0.2 mM	
	NaCl	10.0 mM	
	$CoCl_2\cdot 6H_2O$	0.01 mM	

Table S2. Sequences and functions of primers used in the experiment.

Gene Primer	Sequence of primer (5’-3’)	Use for
TUA5	S: TGCCACCATCAAGACTAAGAGG<br>AS: ACCACCAGGAACAACAGAAGG	qPCR
Pitef	S: GGCCACCGTGACTTTA TCAAGAAC<br>AS: TTCCTTGACGATTTCGTTGAAGCG	qPCR and PCR
ATPase	S:TACACCTCCGTTTCTGCTTCC<br>AS:TGATTTGCATGATTTGTGGGTGG	qPCR
SOS1	S:GGTACTCATCATCGGCTGGG<br>AS: ACCAGGGCCAGCTAGTAAGA	qPCR
NHX2	S:CAACACCATGTCTGGCAGAGATA<br>AS:TGTGTGATTGTATGGTGAGATCCA	qPCR
SOS2	S:GAGGCTTGAAGGCGTTTCTG<br>AS:TTCAGCCTTTCAGTGCTCTTCT	qPCR

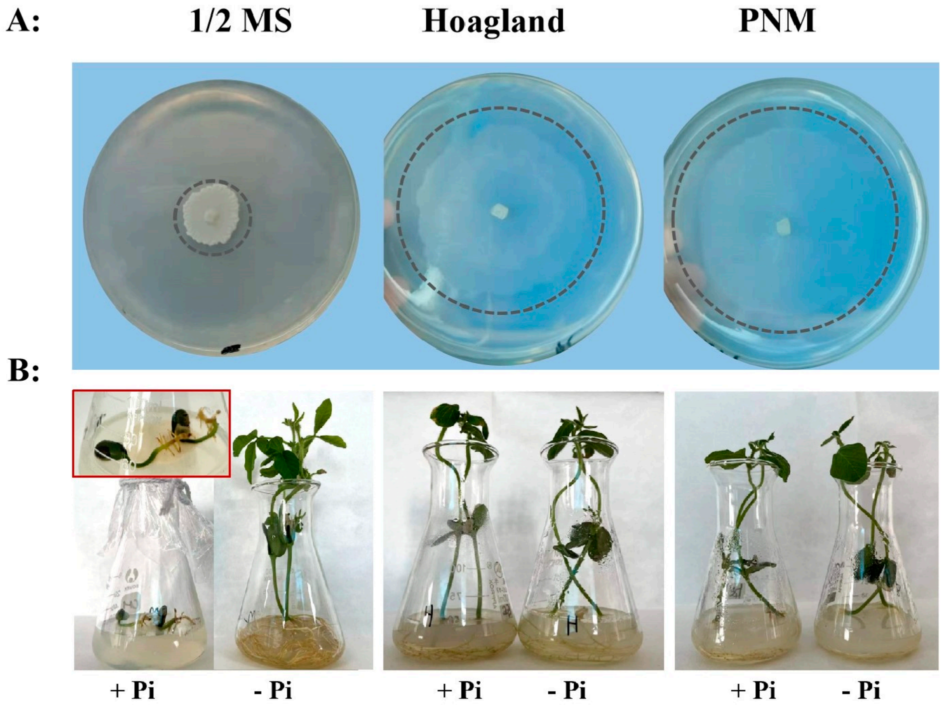

Supplementary Figure S1. The symbiotic between *Piriformis india* and soybean. A: *P. indica* growth in plant medium; B: Symbiotic culture of *P. indica* and soybean seedlings in different medium.
